# Gelsevirine is a novel STING-specific inhibitor and mitigates STING-related inflammation in sepsis

**DOI:** 10.3389/fimmu.2023.1190707

**Published:** 2023-07-31

**Authors:** Yuhong Chen, Huihui Bian, Juan Lv, Wanxue Song, Chunlei Xing, Chunlei Hui, Dinglei Zhang, Chenxi Zhang, Liang Zhao, Yingke Li, Li Su

**Affiliations:** ^1^ School of Pharmacy, Bengbu Medical College, Bengbu, China; ^2^ Institute of Translational Medicine, Shanghai University, Shanghai, China; ^3^ Department of Anesthesiology, Shanghai Changzheng Hospital, Naval Medical University, Shanghai, China; ^4^ Luodian Clinical Drug Research Center, Institute for Translational Medicine Research, Shanghai University, Shanghai, China; ^5^ Department of Pharmacy, Shanghai Baoshan Luodian Hospital, Shanghai, China

**Keywords:** ubiquitination, cecal ligation and puncture, mice, interferon, septic shock

## Abstract

**Background:**

Stimulation of IFN genes (STING) is central to the production of interferon and proinflammatory cytokines in response to microbial DNA or self-DNA in the cytosol. The detrimental role of the activation of STING during sepsis has been well documented.

**Methods:**

Here, we found that gelsevirine (GS) potently inhibit interferon and inflammatory cytokine induction in macrophages exposed to STING agonists (2'3'-cGAMP, IFN stimulatory DNA (ISD), and poly(dA:dT)). I n silico docking analysis and surface plasmon resonance binding study showed that GS bonds with high affinity to the cyclic dinucleotide (CDN)-binding pocket of STING. Biotin pull-down assay also confirmed that GS competitively bonded to STING protein. Furthermore, GS inhibited 2’3’-cGAMP-induced STING dimerization and subsequent activation. In addition, GS induced K48-linked STING ubiquitination and degradation, which was likely through upregulating and recruiting TRIM21. In mice exposed to cecal ligation and puncture (CLP)-induced sepsis, post-operative administration of GS significantly extended the survival period and mitigated acute organ damage.

**Results:**

Overall, GS inhibited STING signaling by competitively binding to the CDN-binding pocket to lock STING in an inactive open conformation, while also promoting K48-linked STING ubiquitination and degradation.

**Conclusions:**

Our findings identify a novel STING-specific inhibitor that could be applied in the treatment of sepsis.

## Introduction

Sepsis is a life-threatening condition characterized by uncontrolled inflammatory responses and multiple organ failures caused by infection (1). Sepsis is a primary global public health problem, giving rise to approximately 11 million deaths annually worldwide and imposing a significant burden on healthcare systems (2). Despite the use of antibiotics and supportive measures, the high morbidity and mortality associated with sepsis remain challenging.

**Figure 1:**
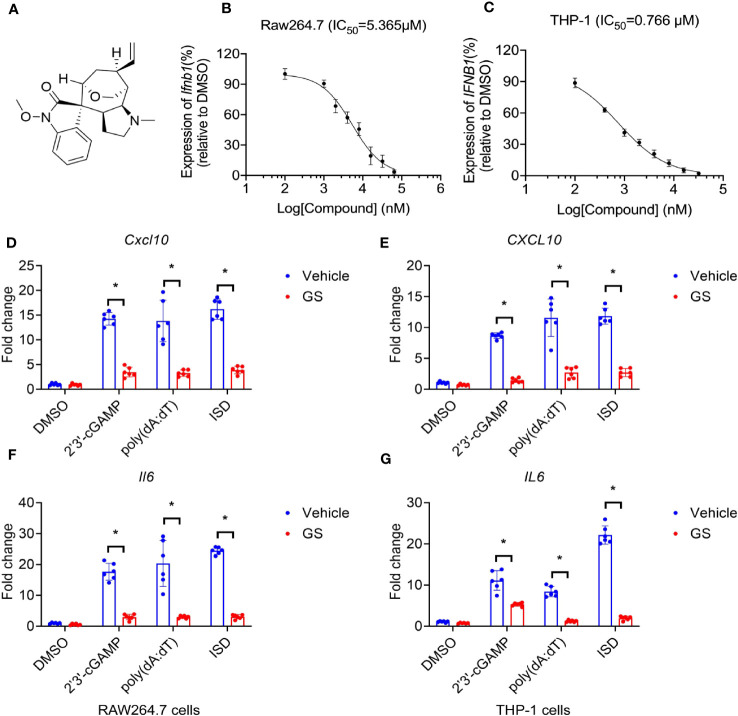
Gelsevirine (GS) inhibits cytosolic DNA-induced expression of interferon and cytokines. Chemical structures of GS **(A)**, and dose-dependent inhibitory curves (b, c, n = 3) a fter pretreatment with different concentrations of GS for 6 hrs, Raw264.7 and THP-1 cells were stimulated by 2’3’-cGAMP (5 μg/ml) for 3 hrs. *Ifnb1* mRNA expression in Raw264.7 cells **(B)** and *IFNB1* mRNA expression in THP-1 cells **(C)** were measured by RT-PCR. Raw264.7 and THP-1 cells were pretreated with GS (10 μM) for 6 hrs and then stimulated with 2’3’-cGAMP (5 μg/ml), ISD (2 μg/ml), or Poly(dA:dT) (5 μg/ml) for 3 hrs. The mRNA expression of *Cxcl10* **(D)** and *Il6* **(F)** in Raw264.7 cells and the mRNA expression of *CXCL10* **(E)** and *IL6* **(G)** in THP-1 cells were measured by RT-PCR. *P < 0.05 vs vehicle group.

Recently, natural compounds from herbal sources have gained attention as a potential novel treatment for septic lethality and sepsis-induced organ dysfunction (3). Gelsevirine (GS, **Figure 1A**) is an alkaloid isolated from Gelsemium elegans Benth (G. elegans), a traditional Chinese herb with various pharmacological properties, including analgesic, anti-inflammatory, and anxiolytic activities (4). Compared with other alkaloids, GS has equally potent anxiolytic activity and less toxicity (5). Our team previously demonstrated that GS inhibited local inflammation and mitigated age-related osteoarthritis (6).

During the initial stages of infection, the innate immune response is initiated by danger-associated molecular patterns (DAMPs) and pathogen-associated molecular patterns (PAMPs) from host and microbial sources (7). The stimulator of interferon genes (STING) detects double-stranded DNA (dsDNA) from infected pathogens and injured host tissues, resulting in the formation of type I interferons (IFNs) and a severe inflammatory response (8). Accumulating evidence suggested that STING signaling plays a detrimental role in lethal sepsis (9–11).

In this study, we investigated whether GS directly targeted STING in inflammatory cells and provided protection in a murine sepsis model established by cecal ligation and puncture (CLP).

## Materials and methods

### Animals

Male C57BL/6J mice (2-month-old) were purchased from Cavens (Changzhou, China). STING-deficient (Tmem173^gt^, Strain #017537) mice (C57BL/6J background) were obtained from Jackson Laboratory (Bar Harbor, ME, USA) and homozygotes did not produce IFN-β in response to cyclic dinucleotides or Listeria monocytogenes infection (12). All the animals were maintained under a 12-hour automated dark-light cycle with a room temperature of 22 ± 2 °C and relative humidity of 50%-60%. Mice were fed *ad libitum* with a standard dry diet and water. All animal protocols were accomplished based on the National Institutes of Health (NIH) guidelines (Guide for the Care and Use of Laboratory Animals) and approved by Shanghai University (ECSHU2021-169).

### Cell culture and transfection

All cell lines were gained from the Cell Bank of the Chinese Academy of Sciences (Shanghai, China). Raw264.7 and THP-1 were maintained with RPMI 1640 containing 10% fetal bovine serum (FBS, Gibco) and 1% Penicillin/Streptomycin (Invitrogen). HEK293T was maintained using DMEM (Invitrogen) supplemented with 10% FBS. Primary hepatocytes, cardiomyocytes, neurons, BMSCs, chondrocytes, and BMMs were isolated from C57BL/6J mice and cultured as described previously (13–18). All cells were maintained at a humid atmosphere of 37°C and 5% CO_2_.

### Surface plasmon resonance binding analysis

The SPR results were obtained using a BiacoreTM T200 machine with CM5 chips (GE Healthcare) at 25°C. The condition of immobilization was optimized by using different concentrations of STING protein with 10 mM sodium acetate (pH 4.0, pH 4.5, pH 5.0). The STING protein was covalently coupled to a CM5 sensor chip, and different concentrations of GS (from 0 to 64 μM) flowed over the Sting chip surface. The experiment was carried out in PBS + 5% DMSO (v/v) buffer system at a flow rate of 30 μl/min. Next, the binding kinetics were analyzed using the software Biacore T200 Evaluation.

### Plasmid construction

Using the plasmid containing the full-length cDNA sequence of human STING (h STING) as a template, the STING CDS sequence was amplified by PCR, EcoRI, and XbaI restriction sites and added at both ends, and 3HA tag coding series was added before XbaI restriction site. The coding sequence was inserted into pcDNA3.0 vector by enzyme digestion. The sequence was confirmed to be identical to the STING transcript (NM_198282.4) in NCBI. The efficiency of plasmid overexpression was verified by HEK 293T cells.

### Immunoprecipitation assay and immunoblot analysis

The cell pellet was obtained and resuspended in RIPA lysis buffer with protease and phosphatase inhibitor cocktail (Sigma) for immunoprecipitation analysis. Cell lysates were incubated with related antibodies (**Table S1**) for 12 hrs at 4°C. Next, protein G agarose beads were added for extra 2 hrs and washed 3 times with lysis buffer. Finally, the samples were boiled with a loading buffer at 100°C for 5 min.

For immunoblot analysis, the immunoprecipitation samples were separated by SDS-PAGE gel and transferred to an NC membrane (Millipore). Immunoblot was performed with related primary and secondary antibodies and visualized by chemiluminescence.

### Synthesis of biotin-GS

Biotin-labeled Gelsevirine was obtained by solid-phase synthesis (**Supplementary Information** (*SI*), **Figure S1**).

#### Fmoc-S5-resin

Dichloromethane (DCM)/ N,N-Dimethylformamide (DMF) mixture solvent was added to resin and swollen for 10 minutes. Then, 3 equivalents of Fmoc-S5-OH and 9 equivalents of N,N-Diisopropylethylamine (DIPEA) in DMF were added to the resin. After 12 hours, the resin was washed with DMF and DCM.

#### Biotin-S5-resin

In total, 20% piperidine in DMF solution was added to remove the Fmoc group for 15 minutes. 3 equivalents of biotin, 3 equivalents of O-(6-Chloro-1-hydrocibenzotriazol-1-yl)-1,1,3,3-tetramethyluronium hexafluorophosphat (HCTU) , and 9 equivalents of DIPEA in DMF were added. After 12 hours, the resin was washed with DMF and DCM.

#### Biotin-S5(Gelsevirine)-resin

0.1 equivalents of the 1st Grubbs' reagent and 3 equivalents of Gelsevirine in DCM solution were added to the resin. After 8 hours of reaction, the resin was washed with DMF and DCM.

#### Biotin-S5(Gelsevirine)-OH

TFE/DCM mixture solution (3:1, v/v/v/v) was added to the resin and the mixture was collected after being stirred for 4 hours. The crude peptides were precipitated by Et_2_O. CH_3_CN/water mixture solution was used to dissolve the resulting residues. It was then analyzed, and purified by RP-HPLC to obtain the target compound Biotin-S5(Gelsevirine)-OH.

### Biotin pulldown assay

HEK293T cells were harvested and lysed in lysis buffer supplemented with a protease inhibitor cocktail. The supernatant was obtained and centrifuged at 1,2000 g for 15 min. Half of the supernatant was incubated in rotation with biotin (5μM) and the other half with biotin-GS (5μM) for 12 hrs at 4°C. Subsequently, the equivalent streptavidin-conjugated agarose beads (Proteintech) that were pre-cleaned by PBS were added and incubated for extra 2 hrs. As described previously, the protein was washed, eluted, separated, and visualized by chemiluminescence.

### STING dimerization assay

Native gel electrophoresis for STING dimerization was identified as described previously (19). Cell lysates containing the native loading buffer were added to the native-PAGE gel and electrophoresed, followed by immunoblot analysis with an anti-STING antibody., followed by immunoblot analysis with an anti-STING antibody.

### Mouse model and Gelsevirine administration

The experimental sepsis mouse model was established in mice through CLP as described previously (20) under anesthesia with 2% isoflurane inhalation (R510-22-16, Shenzhen Ward, China). The sham mice were exposed to an abdominal incision for cecal exposure without ligation and puncture. GS (MUST-19031710, Chegndu Must Bio-Tech, Sicuan, China, purity>99.55%, 10 mg/kg, 20 mg/kg) (6) or astin C (1 mg/kg) (21) was intraperitoneally administered to animals 1 hr before or 5 hrs after CLP surgery. All experiments were carried out abiding by the NIH guidelines (Guide for the Care and Use of Laboratory Animals) and approved by Shanghai University (ECSHU 2021-169).

### Pathology

Sections (3-5 μm) stained with hematoxylin and eosin (H&E) were blindly evaluated by 2 pathologists and scored as described previously (22). The tissue sections of the lungs were stained with antibodies against F4/80 and S1009A to assess the infiltration of macrophages and neutrophils, respectively. Lung injury score: alveolar neutrophil infiltration and aggregation; Pulmonary interstitial neutrophil infiltration; Pulmonary capillary congestion. The alveolar walls were thickened or swollen. Lung injury score: alveolar neutrophil infiltration and aggregation; Pulmonary interstitial neutrophil infiltration; Pulmonary capillary congestion. According to the degree of injury, the score was 0 for no injury, 1 for mild injury, 2 for moderate injury, and 3 for severe injury. The sum of the scores was used as the lung injury score of each group.

### Biochemical analysis

To assess alveolar barrier dysfunction following CLP surgery, protein levels in bronchoalveolar lavage fluid (BALF) were determined with a BCA protein detection kit (#23227, Thermo Fisher Scientific, Waltham, MA, USA).

To assess pulmonary edema, wet weight was taken from the freshly harvested lung tissues. Then, lung tissues were placed on filter paper in an incubator with a set temperature of 80°C for 48 hrs followed by dry weight measurement. Finally, the dry/wet (D/W) ratio of lung tissues was calculated.

The Alanine aminotransferase (ALT) and aspartate aminotransferase (AST) levels in serum were assayed with an automatic biochemical analyzer (Olympus) according to the enzymatic kinetic method. TNF-α (SMTA00B, R&D Systems, Minneapolis, MN, USA) and IL-6 (M6000B, Novus Biologicals, Littleton, CO, USA) in serum and BALF were analyzed by using appropriate ELISA kits. Blood urea nitrogen (BUN) and creatinine in serum were assayed by detection kits (Nanjing Jiancheng Bioengineering Institute).

### Western blot

In brief, protein lysates (25μg) were separated by SDS-PAGE and then transferred by a trans-hybrid turbine transfer system onto a nitrocellulose membrane (Bio-Rad Laboratories, Hercules, CA, USA). Next, 5% BSA buffer was used to block for 4 hrs and incubated with the specific primary antibody at 4 °C for 12 hrs, followed with the appropriate fluorescently labeled secondary antibody for 1 hr at room temperature. Finally, protein bands were shown using the Odyssey Imaging System (LI-COR Biosciences, Lincoln, NE, USA).

### ELISA

CXCL10 and IL6 protein levels in culture supernatants were assayed with an automatic biochemical analyzer (Olympus) according to the enzymatic kinetic method.

### Real-time qPCR measurements

The total RNA of the lungs or cells was isolated with triazole reagent (Thermo Fisher Scientific) based on the manufacturer's protocol. The cDNA was generated from the RNA samples using the Prime foot RTMasterMix (TAKARA Bio Inc., Shiga, Japan). Real-time PCR was carried out by the SYBR green PCR master mixture (Thermo Fisher Scientific) on a Thermal Cycle instrument (Jena Analysis, Germany). The specific primer sequences were shown in **Table S2**.

### Statistical analysis

All data were shown as mean ± standard deviation (SD). One-way ANOVA followed by Student’s t-test was applied to confirm the significance of the difference between results. The survival curve of animal studies and statistics were conducted with the log-rank (Mantel-Cox) test. A difference with a *p*-value < 0.05 was considered to be statistically significant.

## Results

### GS inhibits cytosolic DNA-induced expression of cytokines

T he inhibitory function of GS on the expression of interferon induced by 2’3’-cGAMP (5 μg/ml) was found to be dose-dependent. The IC50 values, representing the half maximal inhibitory concentration, were 5.365 μM and 0.766 μM for Raw264.7 cells (a murine macrophage cell line, **Figure 1B**) and THP-1 cells (a human leukemia monocytic cell line, **Figure 1C**), respectively.

To observe the inhibitory effect of GS on cytokine expression, Raw264.7 cells were pretreated with GS (10 μM) for 6 hours before being stimulated with 2’3’-cGAMP (5 μg/ml), IFN stimulatory DNA (ISD, 2 μg/ml), or Poly(dA:dT) (5 μg/ml) for 3 hours. The mRNA expression of *Cxcl10* (**Figure 1D**) and *Il6* (**Figure 1F**) induced by 2’3’-cGAMP, ISD, and Poly(dA:dT) was inhibited by pretreatment with GS. Similar results were observed in THP-1 cells (**Figure 1E, G**).

Raw264.7 cells and THP-1 cells were pretreated with GS (10 μM) for 6 hours before being stimulated with 2’3’-cGAMP (5 μg/ml), IFN stimulatory DNA (ISD, 2 μg/ml), or Poly(dA:dT) (5 μg/ml) for 24 hours. The expression of Cxcl10 (*SI*, **Figure S2A**) and Il6 (*SI*, **Figure S2C**) induced by 2’3’-cGAMP, ISD, and Poly(dA:dT) was inhibited by pretreatment with GS (23). Similar results were observed in THP-1 cells (*SI*, **Figure S2B, D**).

To assess the cytotoxicity of GS, a CCK8 assay was performed. No remarkable discrepancies were found in the cell viability of RAW264.7(*SI*, **Figure S3A**), THP-1(*SI*, **Figure S3E**), primary cultured cardiomyocytes (*SI*, **Figure S3C**), BMSCs (*SI*, **Figure S3F**), chondrocytes (*SI*, **Figure S3G**), and BMMs (*SI*, **Figure S3H**) after treatment with various concentrations of GS (10-1280 μM) for up to 24 hours. However, GS exhibited robust cytotoxicity to primary cultured hepatocytes (*SI*, **Figure S3B**) and neurons (*SI*, **Figure S3D**) at high doses (>160 μM). These data indicated that GS had good biosafety in the experimental setting(10-160 μM).

### GS binds to the STING and inhibits STING activation

**Figure 2:**
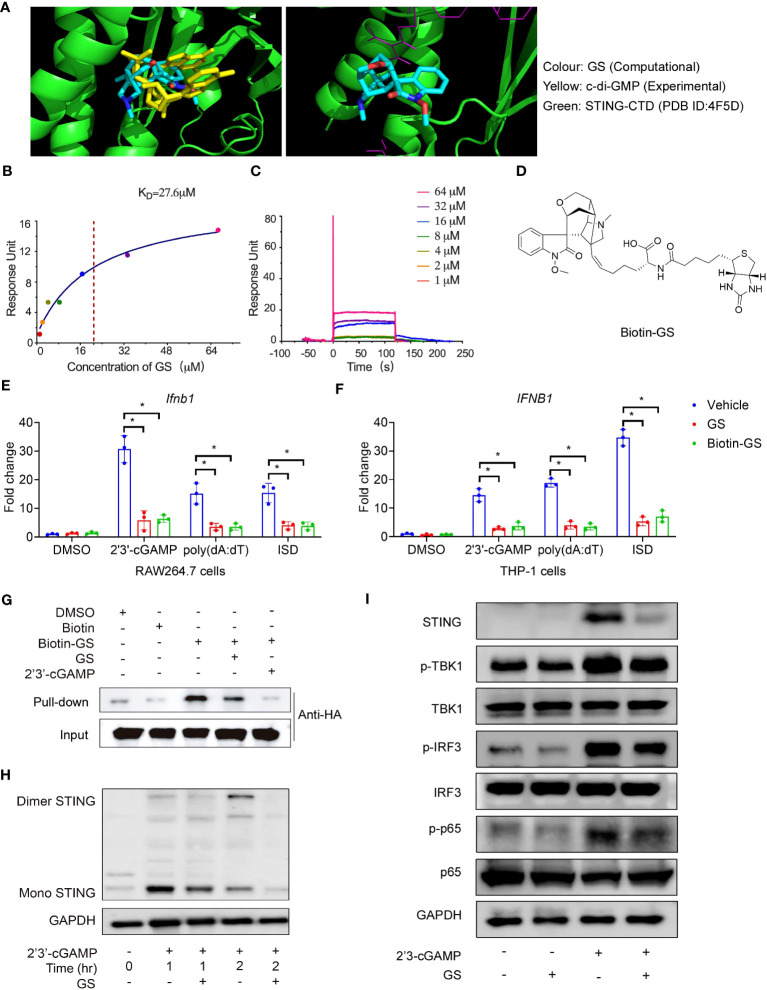
Gelsevirine (GS) binds to the STING and inhibits STING activation. The in silico virtual simulation analysis of the binding between STING-CTD dimer (4F5D) and GS compared with c-di-GMP **(A**, left**)**; the in silico simulation analysis of the binding site of the STING-CTD dimer (4F5D) with GS **(A**, right**)**. Biacore analysis of hSTING-CTD and GS binding. Fitting the binding data to a steady-state 1:1 binding model yielded the binding affinity (Kd) **(B, C)**. Chemical structures of biotin-GS **(D)**. Raw264.7 and THP-1 cells were pretreated with GS (10μM) or biotin-GS (10μM) for 6 hrs and then stimulated with 2’3’-cGAMP (5 μg/ml), ISD (2 μg/ml), or Poly(dA:dT) (5 μg/ml) for 3 hrs. The mRNA expression of *Ifnb1* **(E)** in Raw264.7 cells and mRNA expression of *IFNB1* **(F)** in THP-1 cells were measured by RT-PCR. Cell lysates of HEK293T cells, transfected for 24 hrs with expression plasmids for the HA-tagged STING, were incubated with biotin (5 μM) or biotinylated GS (5 μM) for 1 hr with or without a 10-fold excess (50 μM) of GS or 2’3’-cGAMP (50 μg/ml), followed by pull-down with streptavidin-conjugated beads and immunoblot with anti-HA **(G)**. Raw264.7 cells were pretreated with GS (10 μM) for 6 hrs and then stimulated with 2’3’-cGAMP for 1 hr or 2 hrs. STING dimerization **(H)** was analyzed by immunoblot. Raw264.7 was pretreated with GS (10 μM) for 6 hrs and then stimulated with 2’3’-cGAMP (5 μg/ml) for 3 hrs. The protein expression of STING and phosphorylation of TBK1, IRF3, and p65 was determined by Western blot **(I)**. *P < 0.05 vs vehicle group.

To identify the ability of GS to inhibit STING activation, we used in silico docking technology to analyze the interaction between the human STING C-terminal domain (CTD) (PDB: 4EF5) and GS. The c-di-GMP or 2’3’-cGAMP was centered at the bottom of the cleft formed by the STING-CTD dimer. The in silico simulation showed that GS may be docked in the identical pocket as CDNs (**Figure 2A**). We further assessed the binding affinity of GS to STING using a surface plasmon resonance (SPR) binding study. Results showed that GS had a comparable binding affinity to STING with a Kd value of 27.6 μM (**Figures 2B, C**). To confirm the binding of GS to STING, we synthesized biotinylated GS by chemically attaching biotin to GS (**Figure 2D**). Biotinylated GS exhibited similar inhibitory activities on Ifnb1 (**Figure 2E**) and IFNB1 (**Figure 2F**) expression as GS in Raw264.7 and THP-1 cells, respectively. Furthermore, a biotin pull-down assay confirmed the binding of biotinylated GS to STING. Excess-free GS and 2’3’-cGAMP both competed with biotinylated GS for binding to tagged STING (**Figure 2G**). We also investigated the effect of GS on STING dimerization. Raw264.7 cells were pretreated with or without GS (10 μM) for 6 hrs before being treated with 2’3’-cGAMP for 1 hr or 2 hrs. Treatment with 2’3’-cGAMP (5 μg/ml) enhanced STING dimerization, which was reversed by pretreatment with GS (**Figure 2H**). Raw264.7 cells were pretreated with GS (10 μM) for 6 hrs and then stimulated with 2’3’-cGAMP (5μg/ml) for 3 hrs. We found that GS treatment attenuated phosphorylation of TBK1, IRF3, and p65 (**Figure 2I**) in cells exposed to 2’3’-cGAMP.

### GS promotes the K48-linked ubiquitination and degradation of STING

**Figure 3:**
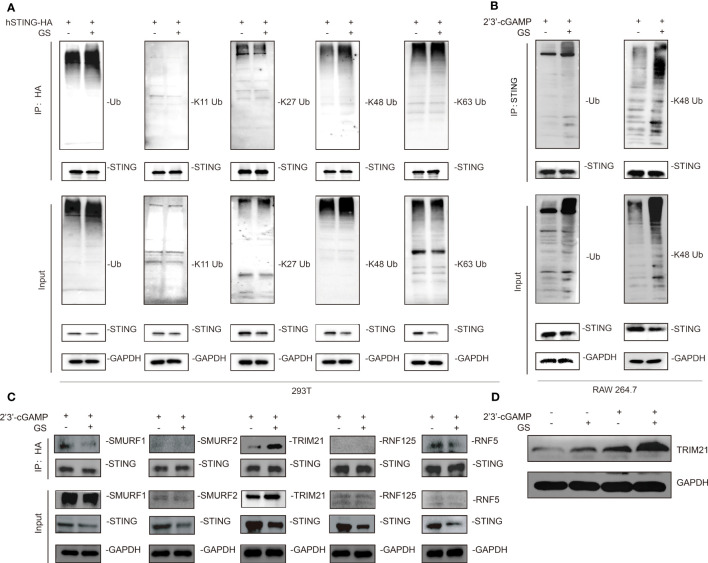
Gelsevirine (GS) promotes K48-linked ubiquitination of STING. HEK293T cells were treated with or without GS (10 μM) for extra 2hrs after transfecting with plasmids expressing STING-HA and UB-flag for 24 hrs. Then, immunoprecipitation (IP) and immunoblot (IB) analysis were carried out **(A)**. Raw264.7 was pretreated with or without GS (10 μM) for 6 hrs and then treated with 2’3’-cGAMP (5 μg/ml) for 3 hrs. Then, IP, and IB analyses were performed **(B)**. HEK293T cells were transfected with plasmids expressing STING-HA for 24 hrs. Next, 10 μM GS was added for 2hrs. Then, IP and IB analyses of SUMRF1, SUMRF2, TRIM21, RNF125, and RNF5 were performed **(C)**. Raw264.7 was pretreated with or without GS (10 μM) for 6 hrs and then activated with 2’3’-cGAMP (5 μg/ml) for 3 hrs. The protein expression of TRIM21 was determined by Western blot **(D)**.

To further probe the mechanism by which GS promoted the degradation of STING, HEK293T cells were transfected with plasmids of STING-HA and UB-flag. After 24 hours, GS (10 μM) and the vehicle was added to the medium for 2 hours. Subsequent immunoprecipitation and western blot results implied that GS did not affect the K11-linked, K27-linked, or K63-linked ubiquitination, but instead increased the K48-linked STING ubiquitination (**Figure 3A**). Furthermore, we demonstrated that GS increased K48-linked STING ubiquitination in Raw264.7 cells exposed to 2’3’-cGAMP (**Figure 3B**).

Based on these findings, we hypothesized that GS might promote the recruitment of a specific E3 ligase to mediate the ubiquitination of STING. We found that GS enhanced the interaction between TRIM21 and STING (**Figure 3C**). Additionally, 2’3’-cGAMP stimulation in Raw264.7 cells increased the protein expression of TRIM21. Notably, GS treatment upregulated TRIM21 expression in both control and 2’3’-cGAMP-stimulated cells (**Figure 3D**).

### GS improves survival and inhibits STING-mediated inflammation in mice with CLP-induced sepsis

**Figure 4:**
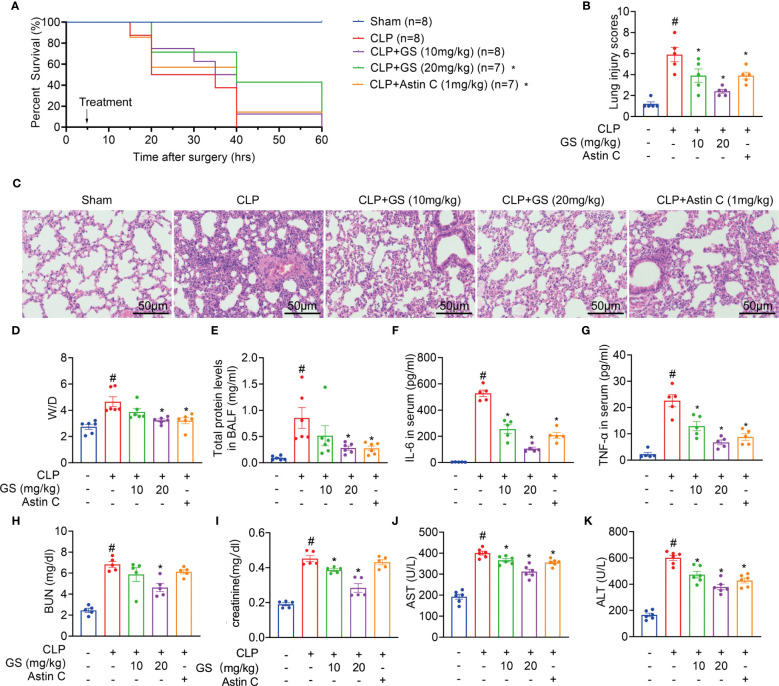
Gelsevirine (GS) improves the survival of mice with CLP-induced sepsis. GS (10, 20 mg/kg) was administrated in 2-month-old C57BL/6J mice 5 hrs after CLP surgery. The survival rate was recorded and calculated **(A)**. 15 hrs after the CLP surgery, the mice were sacrificed, and blood, BALF, and lung tissues were harvested. H&E staining (scale bar = 50 μm) was performed in sections of lung tissues and lung injury was scored **(B, C)**. Graphs showed the Wet-to-dry (D/W) ratios of lung tissues **(D)**, total protein levels in BALF **(E)**, and serum concentrations of IL-6 **(F)**, TNF-α **(G)**, BUN **(H)**, creatinine **(I)**, AST **(J)**, and ALT **(K)**. The data were described as the mean ± SD. #: *P* < 0.05 vs sham group; *: *P* < 0.05 vs CLP group.

GS treatment (10 or 20 mg/kg) was administered to 2-month-old C57BL/6J mice 5 hours after CLP surgery. Astin C, a known inhibitor of STING (21), was used as a positive control drug. Treatment with GS in the CLP group dose-dependently increased the survival rate compared to mice with untreated sepsis (**Figure 4A**).

During sepsis, the lungs are the most vulnerable and critical organs (24, 25). After 15 hours of CLP surgery, the mice were sacrificed, then bronchoalveolar lavage fluid (BALF) and lung tissues were collected. H&E staining results indicated that GS treatment reduced lung injury scores (**Figures 4B, C**). Furthermore, GS treatment dose-dependently decreased the lung wet-to-dry (D/W) ratios (**Figure 4D**) and total protein concentrations in BALF (**Figure 4E**). Our results indicated that GS mitigated sepsis-induced lung impairment in mice.

Treatment of CLP mice with GS dose-dependently reduced the levels of IL-6 and TNF-α in serum (**Figures 4F, G**) and BALF (*SI*, **Figure S5A, B**) and downregulated mRNA expression of *Il6* and *Tnf* (*SI*, **Figure S5C, D**) in lungs.

Moreover, treatment of CLP mice with GS dose-dependently reduced levels of blood urea nitrogen (BUN, **Figure 4H**), creatinine (**Figure 4I**), aspartate aminotransferase (AST, **Figure 4J**), and alanine aminotransferase (ALT, **Figure 4K**) in serum, indicating that GS also mitigated CLP-induced hepatic and renal injuries in mice. Treatment with Astin C reduced ALT and AST levels in serum but had no significant effect on BUN or creatinine levels in serum.

**Figure 5:**
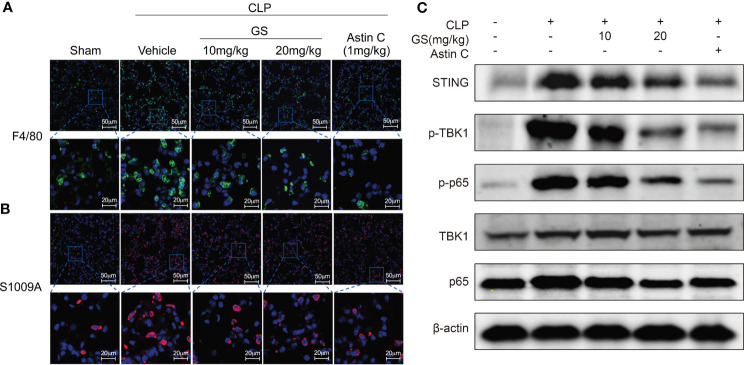
Gelsevirine (GS) inhibits inflammation in the lungs of mice with CLP-induced sepsis. GS (10, 20 mg/kg) was administrated in 2-month-old C57BL/6J mice 5 hrs after CLP surgery. 15 hrs after the CLP surgery, the mice were sacrificed, and lung tissues were harvested. Representative pictures of F4/80 positive cells **(A)** and S1009A positive cells **(B)** in the lungs were shown. The protein levels of STING, phosphorylation of TBK1, and p65 were determined by Western blot **(C)**.

Additionally, GS treatment reduced the number of both F4/80 (**Figure 5A**) and S100A9 (**Figure 5B**) positive cells in the lungs. GS treatment dose-dependently downregulated the expression of STING and suppressed the phosphorylation of TBK1 and p65 (**Figure 5C**), indicating that GS inhibited the STING/TBK1/NF-κB pathway in the lungs of CLP mice. We also found that GS treatment dose-dependently reduced the phosphorylation of IRF3 (SI, **Figure S4A**) and downregulated the mRNA expression of Ifnb1 (SI, **Figure S4B**) in the lungs of CLP mice.

In our study, we further evaluated the protective effects of pretreatment with GS in CLP-induced sepsis in mice. Pretreatment with GS increased the survival rate (*SI*, **Figure S6A**), and reduced lung injury scores (*SI*, **Figure S6B**), D/W ratios (*SI*, **Figure S6C**) of lung tissues, total protein concentrations in BALF (*SI*, **Figure S6D**), and protein levels of IL-6 (*SI*, **Figure S6E**) and TNF-α in serum (*SI*, **Figure S6F**).

### GS does not provide further protection in STING-deficient mice against CLP-induced sepsis

**Figure 6:**
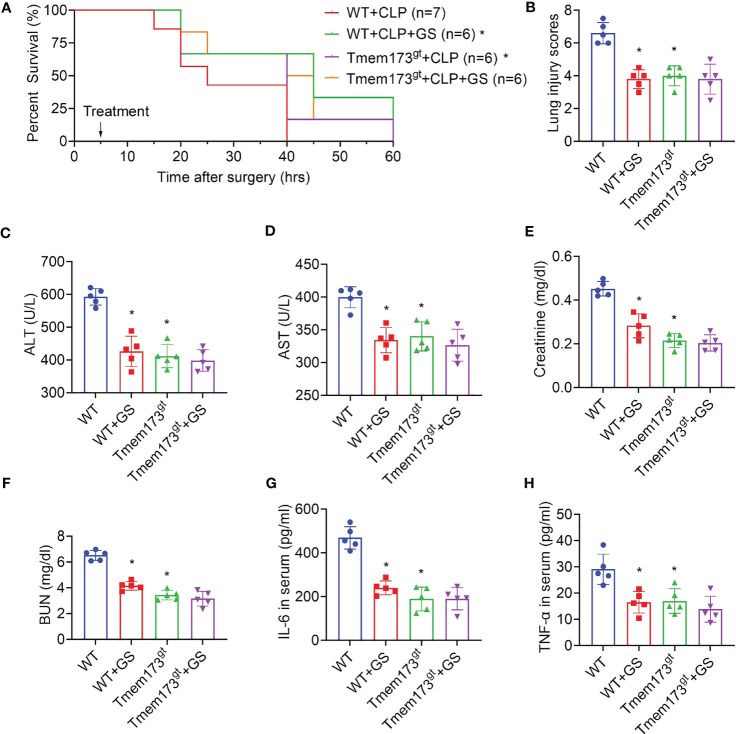
Gelsevirine (GS) does not provide further protection in STING-deficient mice when exposed to CLP. GS (20 mg/kg) was administrated in 2-month-old STING-deficient mice 5 hrs after CLP surgery. The survival rate was recorded and calculated **(A)**. 15 hrs after the CLP surgery, lungs, and blood were harvested. Graphs showed the lung injury scores **(B)** and serum levels of ALT **(C)**, AST **(D)**, creatinine **(E)**, BUN **(F)**, IL-6 **(G)**, and TNF-α **(H)**. *: *P* < 0.05 vs WT group.

Two-month-old STING-deficient and wild-type mice were exposed to CLP surgery and were treated with GS (20 mg/kg) 5 hrs later. We found that STING deficiency alone increased survival rate (**Figure 6A**) and reduced lung injury scores (**Figure 6B**) and levels of ALT (**Figure 6C**), AST (**Figure 6D**), creatinine (**Figure 6E**), BUN (**Figure 6F**), IL-6 (**Figure 6G**), and TNF-α (**Figure 6H**) in serum. Importantly, GS did not provide further protection in STING-deficient mice when exposed to CLP, indicating that the protection of GS against CLP-induced sepsis was dependent on its inhibition of STING.

## Discussion

The findings of the present research highlight the potential of GS as a therapeutic agent for sepsis-induced multiple-organ dysfunction. Sepsis-induced multiple organ dysfunction was a major cause of early death of patients with severe septic shock in ICUs (26). The lungs were particularly susceptible to sepsis-induced damage, and acute lung injury was one of the most common complications of sepsis (27). As key organs in the host-defense action, the liver and kidney also played important roles in sepsis, through the production of acute-phase proteins or cytokines, clearance of the breakdown products of microorganisms and antigens, and metabolic adaptation to inflammatory response (28). In our study, GS treatment significantly improved the impairments in lung, liver, and kidney function in septic mice.

It is noteworthy that the treatment with GS was administered 5 hours after surgery in the CLP mice. Despite the promising results of many therapeutic agents in animal studies, few have shown similar benefits in clinical trials and translated to the clinic (29, 30). In most successful *in vivo* studies, animals usually received agents before or immediately after the septic damages, whereas patients require a definite diagnosis first and are treated after the onset of sepsis. Therefore, GS may hold more promise for translation to a clinical setting.

Our study also identified GS as a potent STING inhibitor that inhibited STING activation in response to cytosolic DNA and dramatically reduced STING-induced gene expression of type I IFNs and proinflammatory cytokines (31). GS was able to compete with cGAMP for binding to the CDN-binding pocket of STING, thereby locking the STING in an inactive conformation.

In comparison, other recently identified STING inhibitors were either inactive (C-176, C-178) or had limited bioactivity against hSTING (NO_2_-FAs, compound 18, Astin C) (21, 32–34). Compound 18 bonds deep in the cleft of the hSTING dimer, but with an IC50 of 11 μM, about 13.75-fold higher than GS (0.766μM) (33). Astin C, with a Kd value of 53 nM, showed higher binding affinity to STING than GS, with a Kd value of 27.6 μM (21). However, the inhibitory effect of astin C (IC50 values of 10.8μM) on intracellular DNA-induced *IFNB* expression was lower than that of GS. That was because GS not only competitively bonded to STING, but also promoted STING ubiquitination and degradation. In the last work, we demonstrated that GS promoted STING K48-linked poly-ubiquitination in chondrocytes (6). In this work, we further found that GS-induced K48-linked STING ubiquitination might be associated with TRIM21. During the host response to viral or bacterial infection, the ubiquitin-proteasome system (UPS) was important in modulating innate immunity and downstream proinflammatory cytokine production (35–37). Tripartite motif (TRIM) proteins, a family of RING-finger ubiquitin E3 ligases, were key regulators in host defenses (38, 39). TRIM21 ubiquitinated and degraded IFI16 and DDX41, two intracellular dsDNA sensors, which triggered downstream STING signaling and innate immunity (40, 41). In addition, TRIM21 downregulated STING protein expression and enhanced the replication of HSV-1 in corneal epithelial cells (31). However, there was no direct evidence that TRIM21 was able to ubiquitinate and degrade STING. Further investigation was required to elucidate the role of TRIM21 in STING ubiquitination.

Taken together, GS inhibited STING signaling by competitively binding to the CDN-binding pocket to lock STING in an inactive open conformation, while also promoting K48-linked STING ubiquitination and degradation. Our findings identify a novel STING-specific inhibitor that could be applied in the treatment of septic shock.

## Data availability statement

The original contributions presented in the study are included in the article/**Supplementary Materials**. Further inquiries can be directed to the corresponding authors.

## Ethics statement

All animal protocols were accomplished based on the National Institutes of Health (NIH) guidelines (Guide for the Care and Use of Laboratory Animals) and approved by Shanghai University (ECSHU2021-169).

## Author contributions

YC: Investigation, Visualization, Conceptualization, Methodology, Data curation, and Writing, original draft. HB: Investigation, Data curation, Formal analysis, Conceptualization, Methodology, and Writing - original draft. JL: Investigation, Data curation, Formal analysis, Conceptualization, and Methodology. CX: Investigation. CH: Investigation. DZ: Investigation. WS: Data curation. CZ: Formal analysis. YL: Investigation, Conceptualization, and Funding acquisition. LZ: Investigation, Conceptualization, and Writing - review & editing. LS: Conceptualization, Supervision, Validation, Writing - review & editing, Project administration, and Funding acquisition. All authors contributed to the article and approved the submitted version.
