# Supplementary material for: Gelsevirine is a novel STING-specific inhibitor and mitigates STING-related inflammation in sepsis
Source: Front Immunol. 2023 Jul 31;14:1190707. doi: 10.3389/fimmu.2023.1190707 (PMC10424845; doi:10.3389/fimmu.2023.1190707)
Supplement: Supplementary file 1 [file DataSheet_1.docx]

Supplementary Material

**Gelsevirine is a novel STING-specific inhibitor and mitigates STING-related inflammation in sepsis**

Yuhong Chen ^1,2†^, Huihui Bian ^2†^, Juan Lv ^2†^, Wanxue Song ^3^, Chunlei Xing ^2^, Chunlei Hui ^2^, Dinglei Zhang ^2^, Chenxi Zhang ^2^, Liang Zhao ^4,5*^, Yingke Li ^3*^, Li Su ^1,2,4*^

^1^ School of Pharmacy, Bengbu Medical College, Bengbu, China

^2^ Institute of Translational Medicine, Shanghai University, Shanghai, China

^3^ Department of Anesthesiology, Shanghai Changzheng Hospital, Naval Medical University, Shanghai, China

^4^ Luodian Clinical Drug Research Center, Institute for Translational Medicine Research, Shanghai University, Shanghai, China

^5^ Department of Pharmacy, Shanghai Baoshan Luodian Hospital, Shanghai, China

Yuhong Chen ^1,2†^, Huihui Bian ^2†^ and Juan Lv ^2†^,These authors contributed equally to this work and share first authorship

*** Correspondence:**Corresponding Author
Li Su (suli1020@shu.edu.cn, Institute of Translational Medicine, Shanghai University, Shangda Road 99, Shanghai, 200444, China), Liang Zhao (zhaoliangphar@163.com, Department of Pharmacy, Shanghai Baoshan Luodian Hospital, Luoxi Road 121, Shanghai, 201908, China) and Yingke Li (liyingke@hotmail.com, Department of Anesthesiology, Shanghai Changzheng Hospital, Naval Medical University, Fengyang Road 415, Shanghai, 200003, China)

Tel: +862166131153; Fax: +862166131153

**Running title**: Gelsevirine and STING in sepsis

Table S1. Key antibodies and chemicals

| **Primary antibodies** | Sources | Identifier | Comments |
| --- | --- | --- | --- |
| STING | Cell Signaling, USA | 13647S | 58μg/ml（1/1000 dilution） |
| p-TBK1 | Cell Signaling, USA | 5483S | 141μg/m（1/1000 dilution） |
| TBK1 | Cell Signaling, USA | 38066S | 20μg/ml（1/1000 dilution） |
| p-IRF3 | Cell Signaling, USA | 37829S | 201μg/ml（1/1000 dilution） |
| IRF3 | Cell Signaling, USA | 6302S | 10μg/ml（1/1000 dilution） |
| p-p65 | Cell Signaling, USA | 3033S | 57μg/ml（1/1000 dilution） |
| P65 | Cell Signaling, USA | 8242S | 208μg/ml（1/1000 dilution） |
| GAPDH | Cell Signaling, USA | 2118S | 42μg/ml（1/1000 dilution） |
| UB | Proteintech, China | 10201-2-AP | 550μg/ml（1/1000 dilution） |
| K48 | Cell Signaling, USA | 8081S | 701μg/ml（1/1000 dilution） |
| K63 | Cell Signaling, USA | 5621S | 371μg/ml（1/1000 dilution） |
| K27 | ABclonal, China | A18202 | （1/1000 dilution） |
| K11 | ABclonal, China | A18197 | （1/1000 dilution） |
| RNF5 | Affinity Biosciences, USA | #DF9490 | 1mg/ml（1/1000 dilution） |
| RNF125 | Affinity Biosciences, USA | #DF4024 | 1mg/ml（1/1000 dilution） |
| TRIM21 | Affinity Biosciences, USA | #DF6717 | 1mg/ml（1/1000 dilution） |
| SMURF1 | Affinity Biosciences, USA | #DF7713 | 1mg/ml（1/1000 dilution） |
| SMURF2 | Affinity Biosciences, USA | #DF 7683 | 1mg/ml（1/1000 dilution） |
| F4/80 | Abcam, USA | Ab6640 | 5μg/ml |
| S100A9 | Abcam, USA | Ab242945 | 1/1000 dilution |
| **Secondary antibodies** |  |  |  |
| Anti-rabbit IgG, HRP-linked Antibody | Cell Signaling, USA | 7074P2 | 60μg/ml（1/1000 dilution） |
| Anti-mouse IgG, HRP-linked Antibody | Cell Signaling, USA | 7076 | 184μg/ml（1/1000 dilution） |
| **Chemicals** |  |  |  |
| 2’3’-cGAMP | APExBIO, USA | B8362 | 5μg/ml |
| Poly(dA:dT) | Invivogen, USA | tlrl-patc | 5μg/ml |
| GS | Chengdu MUST BIO-TECH, China | A1243-250mg | 10μg/ml |
| Astin C | Shyuanye, China | B22233 | 1mg/kg |
| **Elisa Kit** |  |  |  |
| Mouse CXCL10 ELISA Kit | MULTI SCIENCES，China | EK268/2-96 |  |
| Mouse IL-6 ELISA Kit | MULTI SCIENCES，China | EK206/3-96 |  |
| Human CXCL10 ELISA Kit | MULTI SCIENCES，China | EK168-96 |  |
| Human IL-6 ELISA Kit | MULTI SCIENCES，China | EK106/2-96 |  |

Table S2. Sequences of oligonucleotides used as primers

| *Tnf* (mouse) | Sense | CATCTTCTCAAAATTCGAGTGACAA |
| --- | --- | --- |
|  | Antisense | TGGGAGTAGACAAGGTACAACCC |
| *Ifnb1* (mouse) | Sense | AGCTCCAAGAAAGGACGAACAT |
|  | Antisense | GCCCTGTAGGTGAGGGTTGATCT |
| *Il6* (mouse) | Sense | GAGTCCTTCAGAGAGATACAG |
|  | Antisense | TGGTCTTGGTCCTTAGCC |
| *Mx1* (mouse) | Sense | CTGAGATGACCCAGCACCTGAA |
|  | Antisense | CTCCAGGAACCAGCTGCACTTAC |
| *Gapdh* (mouse) | Sense | GACAATTTTGGCATCGTGGA |
|  | Antisense | ATGCAGGGATGATGTTCTGG |
| *CXCL10* (human) | Sense | TGGCATTCAAGGAGTACCTC |
|  | Antisense | TTGTAGCAATGATCTCAACACG |
| *IL6* (human) | Sense | CAGCCCTGAGAAAGGAGACAT |
|  | Antisense | GGTTCAGGTTGTTTTCTGCCA |
| *IFNB1* (human) | Sense | ATTGCCTCAAGGACAGGAG |
|  | Antisense | GGCCTTCAGGTAATGCAGAA |
| *GAPDH* (human) | Sense | CGGAGTCAACGGATTTGGTC |
|  | Antisense | GACAAGCTTCCCGTTCTCAG |

**Figure legend**


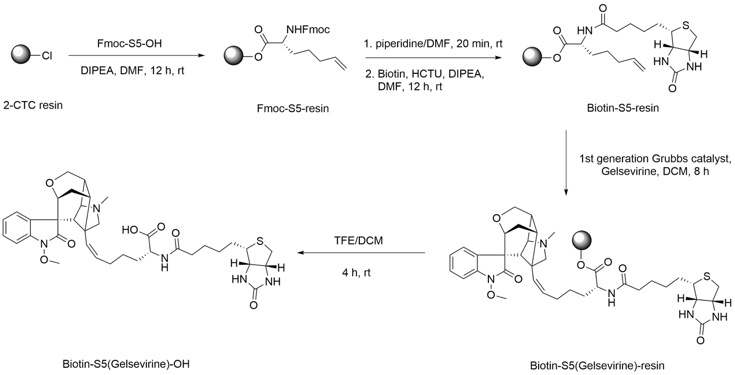


Figure S1. Chemical synthesis of biotin-labeled Gelsevirine


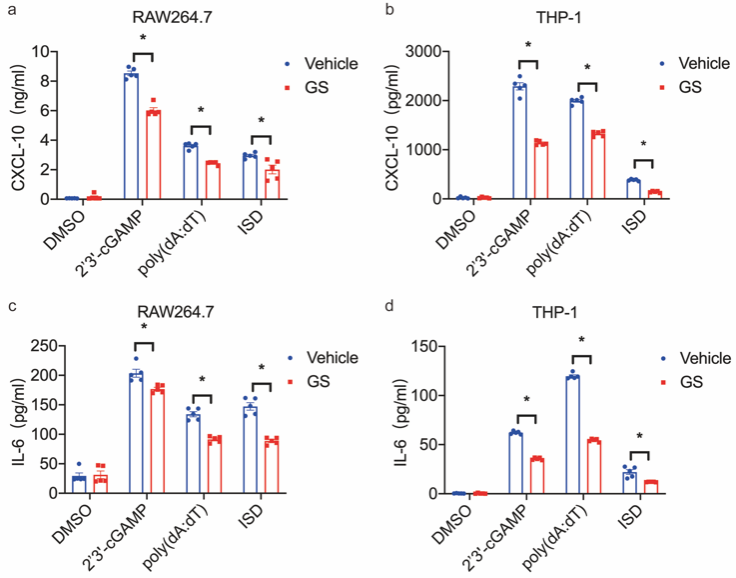


Figure S2. GS inhibits cytosolic DNA-induced expression of cytokines

Raw264.7 and THP-1 cells were pretreated with GS (10 μM) for 6 hrs and then stimulated with 2’3’-cGAMP (5 μg/ml), ISD (2 μg/ml), or Poly(dA:dT) (5 μg/ml) for 24 hrs. The expression of Cxcl10 (A) and Il6 (C) in Raw264.7 cells and the mRNA expression of CXCL10 (B) and IL6 (D) in THP-1 cells were measured by ELISA. *P < 0.05.


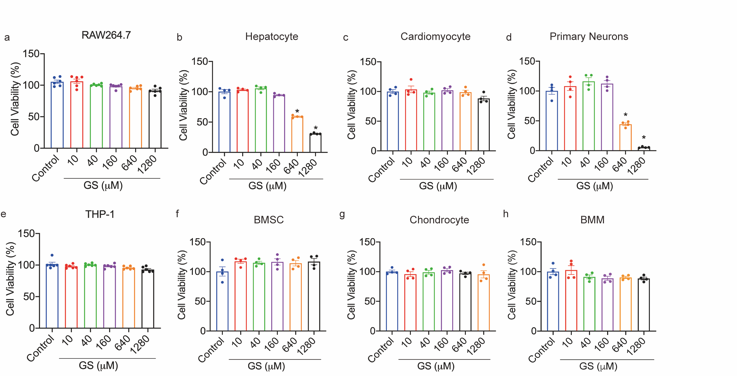


Figure S3. GS has a good biosafety in a variety of primary cells

RAW264.7 (A), THP-1 (E), Primary cultured hepatocytes (B), cardiomyocytes (C), neurons (D), BMSCs (F), chondrocytes (G), and BMMs (H) were isolated from C57BL/6J mice and treated with GS (10, 40, 160, 640, and 1280 μM) for 24 hrs. The cell viability was determined. * P < 0.05 vs Control group.


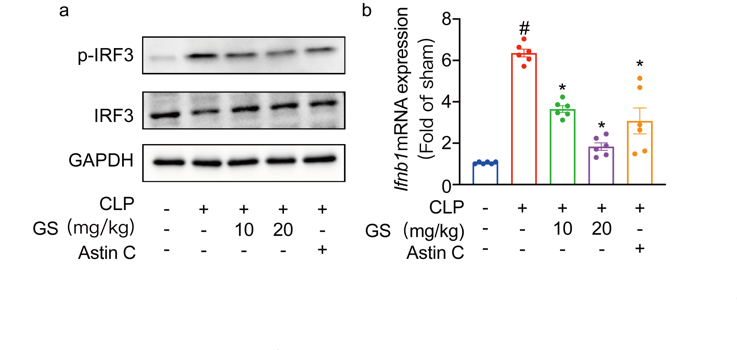


Figure S4. Gelsevirine (GS) inhibits IRF3 pathway in the lungs of mice with CLP-induced sepsis

The protein levels of IRF3, phosphorylated IRF3 and GAPDH were determined by Western blot (A). The mRNA expression of Ifnb1(B) in lung was measured by RT-PCR. #P < 0.05 vs sham group; *P < 0.05 vs CLP group.


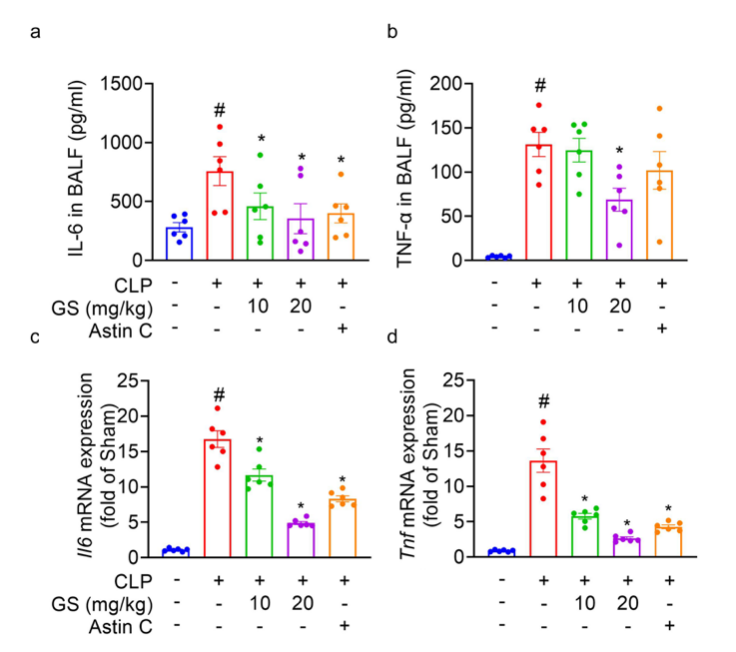


Figure S5. GS inhibits inflammation in lungs of mice with CLP-induced sepsis

GS (10, 20 mg/kg) was administrated in 2-month-old C57BL/6J mice 5 hrs after CLP surgery. 15 hrs after the CLP surgery, the mice were sacrificed, and BALF and lung tissues were harvested. Graphs showed the protein levels of IL-6 **(A)** and TNF-α **(B)** in BALF and mRNA expression of *Il6* **(C)** and *Tnf* **(D)** in the lungs. #*P* < 0.05 vs sham group; **P* < 0.05 vs CLP group.


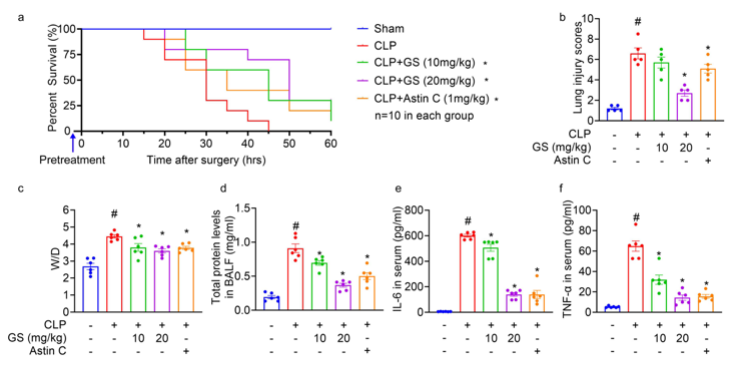


Figure S6. Pretreatment with GS improves survival of mice with CLP-induced sepsis

GS (10, 20 mg/kg) was administrated in 2-month-old C57BL/6J mice an hour before CLP surgery. The survival rate was recorded and calculated **(A)**. 15 hrs after the CLP surgery, the mice were sacrificed, and blood, BALF, and lung tissues were harvested. Lung injury was scored **(B)**. Graphs showed the Wet-to-dry (W/D) ratios of lung tissues **(C)**, total protein levels in BALF **(D)**, and serum levels of IL-6 **(E)** and TNF-α **(F)**. #*P* < 0.05 vs sham group; **P* < 0.05 vs CLP group.
